# Supplementary material for: Content-rich biological network constructed by mining PubMed abstracts
Source: BMC Bioinformatics. 2004 Oct 8;5:147. doi: 10.1186/1471-2105-5-147 (PMC528731; doi:10.1186/1471-2105-5-147)
Supplement: Additional File 2 — The original results of the above study (non-essential files are deleted to keep the file size under the limit set by BMC bioinformatics). [file 1471-2105-5-147-S2.bz2 › chilibotAdditionalFile2/dip10/692ID12004122E18014/html/BTUC_BTUD.html]

 


 **BTUC** and **BTUD** 
  
Found 7 abstracts in PubMed, retrieved 7.  
 

 What does Google say? 
 PDF only 
| .edu only 

---

**Interactive relationship** (e.g. stimulation, inhibition, etc)

**Neutral relationship**- The apparent molecular weight of the  **btuC**  product was approximately 2 00, that of the  **btuD**  product was 2 00.  Ref: 3528128 J Bacteriol, 1986
- The two ATP binding cassettes  **BtuD**  are in close contact with each other, as are the two membrane spanning subunits  **BtuC** .  Ref: 12004122 Science, 2002

**Non-interactive relationship** (e.g. studied together, co-existance, homology, etc.)

- The  **BtuC**  subunits provide 20 transmembrane helices grouped around a translocation pathway that is closed to the cytoplasm by a gate region whereas the dimer arrangement of the  **BtuD**  subunits resembles the ATP bound form of the Rad50 DNA repair enzyme.  Ref: 12004122 Science, 2002
- The relative production of BtuE and  **BtuD**  in response to plasmids carrying transposon insertions suggested that the three genes are arranged in an operon in the order  **btuC**  btuE  **btuD** .  Ref: 3528128 J Bacteriol, 1986
- The growth response to CN Cbl of the btuF mutant was much stronger than the slight impairment previously described for  **btuC** ,  **btuD** , or btuF mutants.  Ref: 11790740 J Bacteriol, 2002
- The gene order in this portion of the chromosome map was found to be pps zdh 3 Tn10  **btuD**   **btuC**  pheS.  Ref: 2987192 J Bacteriol, 1985
- Transport of vitamin B12 across the cytoplasmic membrane of Escherichia coli requires the products of  **btuC**  and  **btuD** , two genes in the btuCED operon.  Ref: 2671656 Mol Gen Genet, 1989
- In addition to this plasmid with a 6 kilobase insert of S. typhimurium DNA, vitamin B12 utilization by E. coli btuB strains required the  **btuC**  and  **btuD**  products, necessary for transport across the cytoplasmic membrane, but not the btuE or tonB product.  Ref: 2656634 J Bacteriol, 1989
- Hence, null mutations in  **btuC**  and  **btuD**  were constructed and revealed that the  **btuC**  mutant had a strong impairment similar to that of the btuF mutant.  Ref: 11790740 J Bacteriol, 2002
- Transport across the cytoplasmic membrane CM requires the  **BtuC**  and  **BtuD**  proteins, which are most related in sequence to the transmembrane and ATP binding cassette proteins of periplasmic permeases for iron siderophore transport.  Ref: 11790740 J Bacteriol, 2002
- Mutations in  **btuC**  and  **btuD**  are complemented by recombinant plasmids carrying a 10 base pair HindIII HincII DNA fragment.  Ref: 3528128 J Bacteriol, 1986
- Transposon insertions in the region between  **btuC**  and  **btuD** , as well as those in the two genes, conferred a deficiency in vitamin B12 utilization and transport when they were crossed onto the chromosome.  Ref: 3528128 J Bacteriol, 1986
- Since the vitamin accumulated in  **btuC**  or  **btuD**  mutants is readily released from the cell by chase or osmotic shock and does not undergo the usual metabolic conversions, the products of these genes might participate in transport across the cytoplasmic membrane.  Ref: 3528128 J Bacteriol, 1986
